# Determinants of Unpaid Hospital Charges Among Non-Resident Foreign Patients: A Retrospective Single-Center Study in Tokyo, Japan

**DOI:** 10.3390/healthcare13222893

**Published:** 2025-11-13

**Authors:** Soichiro Saeki, Yukiko Nakamura, Nanako Miki, Yasuyo Osanai, Mayumi Horikawa, Chihaya Hinohara

**Affiliations:** 1 International Health Care Center, National Center for Global Health and Medicine, Japan Institute for Health Security, Tokyo 162-8655, Japan; 2 Emergency Medicine and Critical Care, National Center for Global Health and Medicine, Japan Institute for Health Security, Tokyo 162-8655, Japan; 3 Division of Public Health, Department of Social Medicine, Graduate School of Medicine, The University of Osaka, Osaka 565-0871, Japan; 4 Department of Medical Administration, National Center for Global Health and Medicine, Japan Institute for Health Security, Tokyo 162-8655, Japan; 5 Department of Nursing, National Center for Global Health and Medicine, Japan Institute for Health Security, Tokyo 162-8655, Japan

**Keywords:** travel health, migrant health, global health, travel insurance, payment, foreigners, universal health coverage, foreign patients, international patients

## Abstract

**Background/Objectives**: Unpaid medical expenses incurred by foreign nationals represent a growing concern for healthcare systems amid increasing international mobility. Japan, which lacks mandatory public insurance coverage for non-resident visitors, faces particular vulnerability in terms of uncompensated hospital care. This study aims to identify factors contributing to unpaid medical charges among uninsured, non-resident foreign patients hospitalized at a tertiary care facility in Tokyo. **Methods**: This retrospective observational analysis was conducted using medical and administrative data from patients admitted between January 2023 and February 2025. Patients who received elective medical tourism care were excluded. Data on demographics, length of hospital stay, care intensity, payment status, and third-party financial assistance were analyzed. Logistic regression models were applied to assess predictors of nonpayment. **Results**: Among 153 eligible cases, 9 patients (5.9%) had outstanding hospital bills upon discharge. Compared with those with completed payments, the unpaid group experienced longer admissions, more intensive care utilization, and higher total charges. Notably, the absence of third-party financial support (primarily travel insurance) was significantly associated with unpaid charges. Multivariate analysis identified this factor as the main independent predictor (adjusted odds ratio [OR]: 0.12; 95% confidence interval [CI]: 0.02–0.915; *p* = 0.040). Total amount of billing was also statistically significant (adjusted odds ratio [OR]: 1.01; 95% confidence interval [CI]: 1.00–1.01; *p* = 0.039). **Conclusions**: These findings highlight the importance of private insurance in mitigating financial risk in hospitals. Implementing policy measures to promote or require insurance enrollment, along with streamlined reimbursement systems, may contribute to sustainable care delivery for international patients.

## 1. Introduction

In recent decades, the globalization of travel and commerce has led to a substantial increase in international mobility [1]. While this trend has fostered economic and cultural exchanges, it has also introduced complex challenges for healthcare systems worldwide [2,3,4,5]. One such challenge is the provision of timely, equitable, and financially sustainable care for foreign nationals who become ill while abroad. Notably, an estimated 40–80% of international travelers experience some form of illness during or shortly after their journeys, underscoring the global relevance of this issue [6].

Japan, as a major international travel destination, has witnessed a sharp resurgence in inbound tourism since the COVID-19 pandemic [7]. In response, healthcare institutions have been compelled to adapt to the evolving demands of a globally mobile patient population [8]. Despite efforts to improve accessibility, such as promotion of the usage of multilingual signage, linguistic assistance such as healthcare interpreting and accreditation of foreigner-friendly hospitals [9,10,11], Japan’s healthcare system remains insufficiently prepared to meet the diverse needs of non-resident foreign patients [12,13,14]. Language barriers, cultural differences, limited health literacy, and unfamiliarity with Japan’s medical payment system continue to hinder effective care delivery [15].

Among the emerging concerns is the growing incidence of unpaid medical charges incurred by non-resident patients [14]. Unpaid medical expenses impose significant financial pressure on hospitals, particularly those providing emergency or tertiary care. Uncompensated care contributes to resource depletion, reduces hospitals’ capacity to maintain international patient services, and may indirectly affect equity in access to emergency care. A national survey reported that 18.3% of Japanese hospitals had experienced cases of unpaid medical bills from foreign patients [16], with tertiary care institutions bearing a disproportionate burden [17]. These unpaid expenses place substantial fiscal strain on hospitals, particularly in the absence of standardized cross-border billing systems and third-party reimbursement mechanisms. Japan’s social health insurance system provides universal coverage for citizens and foreign residents [18,19], limiting out-of-pocket costs to 10–30% or 0% for welfare recipients [20,21]. However, non-resident travelers are excluded from this system [22,23] and are therefore fully responsible for their medical expenses [24]. This financial liability, combined with the inherently transient nature of international travel, frequently results in uncollected payments, placing substantial fiscal strain on healthcare institutions. The absence of standardized cross-border billing systems and third-party reimbursement mechanisms further impedes cost recovery. Should the patient require medical evacuation or repatriation, the economic burden increases substantially [25], further exacerbating the financial vulnerability of the individual [24,26].

In other countries, such as the United States and Germany, previous studies have identified insurance status and socioeconomic factors as key predictors of unpaid hospital bills, particularly in emergency care settings. A study from the United States has shown that lack of insurance is a key predictor of unpaid hospital bills in emergency care settings, often leading to significant uncompensated costs for physicians and hospitals [27]. Similarly, research from Germany highlighted the systemic challenges posed by uninsured migrants requiring hospital care [28]. While these findings resonate with the Japanese situation, Japan’s healthcare system presents a unique case where universal coverage is guaranteed for residents but not extended to short-term visitors.

However, Japan-specific data, particularly regarding non-resident foreign patients, remain scarce. Although several studies have previously reported unpaid medical bills by foreign patients in Japan [29,30,31,32,33], none have empirically examined their determinants using hospital-based patient-level data. This absence of quantitative evidence has hindered the development of targeted countermeasures. Therefore, this study aims to fill this gap by identifying the factors associated with unpaid hospital charges in Japan’s largest tertiary emergency hospital for foreign patients.

## 2. Materials and Methods

This single-center retrospective observational study was conducted at the National Center for Global Health and Medicine (NCGM, Shinjuku, Tokyo, Japan), a tertiary emergency and critical care center located in Tokyo, Japan. NCGM is a hospital of the Japan Institute for Health Security (JIHS), priorities explicitly include “human resource development and international cooperation,” focusing on building expertise and fostering partnerships with other countries and global organizations [34]. NCGM has an International Health Care Center (ICC) that aids patients from abroad when receiving medical care [35] and a high-volume emergency department with approximately 20,000 patient visits per year [36], of which approximately half are emergently transported [36,37], accepting many foreign patients in emergency situations.

The study was approved by the institutional ethics committee (approval number: JIHS-S-004951-01) and was conducted in accordance with the Declaration of Helsinki. Informed consent was obtained using an opt-out approach via the hospital website.

### 2.1. Participants

All non-resident foreign patients admitted to our hospital between January 2023 and February 2025 were automatically identified through the electronic medical record and hospital billing database. Because all eligible cases were included, no additional sampling or random selection was performed. This study therefore represents a complete enumeration of all non-resident foreign inpatients during the study period.

Patients admitted for elective procedures or medical tourism were not included from this study. Non-residency was defined as individuals initially staying in Japan without residency visas.

### 2.2. Data Collection

Administrative and clinical data were extracted from hospital databases and electronic medical records. The following variables were collected: demographics, reason for visiting Japan (e.g., tourism, visiting family, business), length of hospital stay, visa status (Temporary visit or official), days of high-acuity units (days spent in the intensive care unit (ICU), high care unit (HCU), stroke care unit (SCU), emergency medicine and critical care unit), total billed amount (in Japanese Yen, JPY), third-party financial support, death upon discharge, and unpaid charge status and amount at discharge.

Unpaid medical charges were defined as any hospital bill with no recorded payment at the time of discharge. Third-party financial support was defined as financial assistance from sources other than the patient’s family or friends, including private travel insurance, support under the public subsidies for certain infectious diseases in Japan [38], or support from refugee aid groups, including the Refugee Assistance Headquarters (RHQ).

### 2.3. Statistical Analysis

The primary outcome was the presence or absence of unpaid hospital charges at discharge. The dependent variable was coded as a binary indicator: unpaid = 1, paid = 0. The variables included in the model were: age, region of origin, length of stay, high-acuity unit days, purpose of visit, residence status, international repatriation, and third-party financial support.

Unpaid charges were coded as 1 and included both full and partial non-payments, defined as cases where complete settlement had not been recorded by the time of discharge. Paid cases (coded as 0) indicated full payment by either the patient or a third-party sponsor. Outcome classification was based on the hospital’s electronic billing system, which records payments by patients, insurance companies, and other third-party sponsors.

Univariate analyses were performed using Fisher’s exact test for categorical variables and the Mann–Whitney U test for continuous variables. Multivariate logistic regression was conducted to identify the independent predictors of unpaid charges. As the outcome variable (unpaid vs. paid hospital charges) was binary, logistic regression was used to estimate the odds of nonpayment associated with each independent variable. Because only a few events were observed, the possibility of the logistic regression model being prone to overfitting was of concern; hence, covariate selection was restricted to the minimally sufficient adjustment set determined by a Directed Acyclic Graph (DAG), to preserve conceptual validity while minimizing model complexity (see Supplementary Materials). In addition, to adjust for overfitting, the region and reason for visit to Japan were rounded to “visiting family”, “tourism”, and “others (including the rest of the cases)”, and “Asia (including Southeast Asia, East Asia, and Japan)” and “others (including the rest of the cases)”.

All analyses were performed using EZR (Saitama Medical Center, Jichi Medical University), a graphical interface for R [39]. version 4.3.1 (R core team, Vienna, Austria) [40]. Statistical significance was set at *p* < 0.05.

## 3. Results

### 3.1. Patient Characteristics

A total of 153 non-resident foreign patients met the inclusion criteria. Among them, nine patients (5.9%) had unpaid hospital charges at the time of discharge. Compared with patients who completed payment, those with unpaid charges had significantly longer hospital stays (median 12 days vs. 6 days, *p* = 0.027) and higher total billed amounts (median 4,538,320 JPY vs. 1,091,270 JPY, *p* = 0.008). The proportion of patients who received third-party financial support was significantly lower in the unpaid group (11.1%) compared to the paid group (61.0%; *p* = 0.004). Table 1 summarizes the baseline characteristics of the study population.

### 3.2. Logistic Regression Analysis

The univariate analysis identified the absence of third-party financial support as a significant factor associated with unpaid charges. In the multivariate logistic regression model, while third-party financial support remained a statistically significant independent predictor of payment (adjusted odds ratio [OR]: 0.12; 95% confidence interval [CI]: 0.02–0.915; *p* = 0.040), the total billed amount was statistically significant as a risk of non-payment (adjusted odds ratio [OR]: 1.01; 95% confidence interval [CI]: 1.00–1.01; *p* = 0.039). Other variables, including length of hospital stay, purpose of visit, and region of origin, did not retain statistical significance after adjustment. Detailed results of the regression analysis are presented in Table 2.

## 4. Discussion

This study identified the absence of third-party financial support, particularly private travel insurance, as the strongest independent predictor of unpaid hospital charges among non-resident foreign patients in Japan. Although the amount of billing also became statistically significant, the OR was as significant. These findings are consistent with previous international studies where lack of insurance coverage has been repeatedly identified as the strongest predictor of unpaid hospital bills in emergency settings from abroad [27,28], These parallels suggest that the mechanisms underlying unpaid care—limited insurance coverage and transient residency—transcend national boundaries. While factors such as length of hospital stay and purpose of visit showed associations in the univariate analysis, they did not retain significance after adjustment. This finding suggests that financial coverage, rather than clinical severity or travel intent, is the primary determinant of payment outcomes in this population.

In Japan, where non-resident travelers are excluded from the social health insurance system [18,22], uninsured foreign patients, especially those requiring high-acuity care, pose a substantial financial risk to hospitals. Our findings underscore the importance of policy-level interventions for mitigating this risk. Measures such as mandating proof of travel insurance upon entry or incentivizing insurance enrollment through visa processes may help reduce the incidence of unpaid charges.

However, despite ongoing efforts by the Japan Tourism Agency, the proportion of foreign tourists purchasing travel insurance has remained stagnant at approximately 73% over the past six years [41]. This suggests that the current promotional strategies may be insufficient. Notably, previous studies have identified tourists visiting friends and relatives (VFRs) as a subgroup with particularly low insurance uptake and heightened vulnerability to health risks [42,43]. Cultural differences in perceptions of insurance and healthcare utilization may further explain the low uptake of travel insurance among certain groups of travelers, suggesting a need for culturally tailored informational campaigns. Targeted outreach to this population, through consular services, travel agencies, or community organizations, may be an effective strategy to improve coverage rates.

Beyond individual behavior, systemic reforms are needed. Mandating travel insurance for short-term visitors, establishing centralized reimbursement systems, and streamlining communication between hospitals and insurers could reduce administrative burden and financial losses. These measures would not only protect healthcare institutions but also promote equitable access to emergency care for international travelers.

As Japan continues to position itself as a global destination, addressing the financial risks associated with cross-border care will be essential for ensuring both sustainability and fairness in its healthcare system.

While our data derive from a single Japanese tertiary hospital, the issue of unpaid hospital charges among non-resident patients is not unique to Japan. Similar challenges have been reported in the United States, Germany, Switzerland, and other high-income countries, where hospitals struggle to recover costs for uninsured or transient foreign patients [25,27,28]. From a theoretical standpoint, insurance operates as a precommitment mechanism that ensures traceable billing and reduces the behavioral uncertainty of ad hoc payment. However, the magnitude of this effect may vary across institutional settings, tourist profiles, and cost recovery systems.

What makes the Japanese case distinctive is the coexistence of universal health coverage for residents with the complete exclusion of short-term visitors from this system, offering insights into how gaps emerge even in settings with robust insurance schemes. Therefore, our findings not only provide preliminary domestic evidence but also contribute to the broader international discourse on cross-border health financing. Strengthening mechanisms for international cost recovery and promoting insurance uptake may have relevance well beyond Japan.

Our findings must be interpreted with caution, as the limited number of unpaid cases raise concerns regarding statistical stability and potential overfitting. The wide confidence intervals and limited number of outcome events highlight potential model instability. These results should be regarded as exploratory and interpreted cautiously, while the consistency of the associations with previous reports lends some external credibility. Nonetheless, given that our hospital admits the largest number of foreign patients in Japan, this cohort provides valuable preliminary evidence that underscores the importance of third-party financial support in preventing unpaid charges. Future multicenter or national registry studies are essential to validate and expand upon these results.

### Limitations

This study has several limitations that should be acknowledged. First, it was conducted at a single tertiary care institution in Tokyo, which may limit the generalizability of the findings to other healthcare settings or regions within Japan. The patient population and institutional policies at this facility may not reflect those of smaller hospitals or rural areas.

Second, although we used multivariable logistic regression to adjust for potential confounders, the number of outcome events was limited to only nine unpaid cases. This inevitably raises concerns regarding model overfitting and the statistical stability of the estimated odds ratios [44]. To mitigate this limitation, we employed a directed acyclic graph (DAG) to identify a minimally sufficient adjustment set, thereby prioritizing conceptual validity over model parsimony alone. Nevertheless, the small number of events may still compromise the robustness of our estimates, and our findings should be interpreted as exploratory and hypothesis-generating. Importantly, as our hospital is one of the leading tertiary emergency centers in Japan and admits the largest number of foreign patients nationwide [35], this cohort represents the most comprehensive dataset currently available on this issue. We believe that presenting these exploratory results provides valuable preliminary evidence and highlights the urgent need for larger multicenter registry studies to confirm and extend our findings.

Third, the operational definition of third-party financial support encompassed a heterogeneous group of sources, including private travel insurance, public subsidies for infectious diseases, and support from humanitarian organizations. Owing to the small number of unpaid cases, we were unable to perform subgroup analyses to assess the relative impact of each type of support.

Fourth, given that our data originates from a tertiary emergency hospital serving predominantly short-term visitors, caution is warranted in generalizing these results to elective care settings or different seasonal patterns of tourism.

Finally, as with all retrospective studies relying on administrative and electronic medical records, there is a risk of data entry errors, missing information, or misclassifications. These factors may have influenced the accuracy of the findings and should be considered when interpreting the results.

## 5. Conclusions

Unpaid hospital charges among non-resident foreign patients remain a growing concern in Japan’s healthcare system. This study identified the absence of third-party financial support as the main determinant of nonpayment. Although limited by the small sample size and single-center design, these exploratory findings highlight the importance of strengthening travel insurance coverage and establishing international cost-recovery frameworks to ensure the financial sustainability of cross-border healthcare.

These findings highlight the urgent need for systemic measures to mitigate financial risk for healthcare institutions. Strengthening insurance requirements for international visitors, promoting the broader uptake of travel insurance, and establishing efficient reimbursement mechanisms may help ensure the sustainability of cross-border medical care.

As Japan continues to position itself as a global destination, both for tourism and international cooperation, addressing the financial vulnerabilities associated with foreign patient care will be essential. Future multicenter studies with larger sample sizes are warranted to validate these findings and inform national policy development.

In conclusion, while limited by sample size and single-center design, our findings suggest that lack of third-party financial support strongly correlates with unpaid hospital bills. These exploratory results underscore the importance of cross-border insurance mechanisms and call for multicenter registry studies to validate these patterns.

## Figures and Tables

**Table 1:** Background Characteristics of the Patients.

		Unpaid at Discharge (=1)	Fully Paid at Discharge (=0)	*p*
*n*		9	144	
International Repatriation (%)	Yes	1 (11.1)	2 (1.4)	0.167
	No	8 (88.9)	142 (98.6)	
Death upon discharge (%)	Yes	1 (11.1)	7 (4.9)	0.392
	No	8 (88.9)	137 (95.1)	
Age (median [IQR])		35.00 [31.00, 48.00]	48.50 [30.00, 64.25]	0.169
Hospital Length of stay (median [IQR])		12.00 [8.00, 14.00]	6.00 [3.00, 10.25]	0.027 *
High-acuity unit days (median [IQR])		8.00 [5.00, 12.00]	4.00 [2.00, 8.00]	0.156
Fiscal Year (%)	FY2022	1 (11.1)	5 (3.5)	0.287
	FY2023	3 (33.3)	72 (50.0)	
	FY2024	5 (55.6)	67 (46.5)	
Billed amount JPY per ¥10,000 (median [IQR])		453.8 [220, 633]	109.1 [67, 180]	0.004 *
Reason for visit to Japan (%)	Immigration detention	0 (0.0)	1 (0.7)	0.313
	Overstay	0 (0.0)	1 (0.7)	
	Visiting Family	2 (22.2)	12 (8.3)	
	Residency (e.g., embassy employees)	0 (0.0)	9 (6.2)	
	Work/Training/Study	1 (11.1)	13 (9.0)	
	For treatment	0 (0.0)	1 (0.7)	
	For treatment at another medical facility	0 (0.0)	2 (1.4)	
	Refugee	1 (11.1)	2 (1.4)	
	Tourism	5 (55.6)	103 (71.5)	
Visa status (%)	Other	0 (0.0)	2 (10.5)	1
	Not applicable	0 (0.0)	1 (5.3)	
	Official	0 (0.0)	1 (5.3)	
	Temporary Visit	3 (100.0)	15 (78.9)	
Region (%)	Other	3 (33.3)	19 (13.2)	0.224
	Europe/Americas/Oceania	2 (22.2)	74 (51.4)	
	East Asia	3 (33.3)	30 (20.8)	
	Southeast Asia	1 (11.1)	18 (12.5)	
	Japan	0 (0.0)	3 (2.1)	
Third-party financial support (e.g., travel insurance, public subsidy) (%)	No	6 (66.7)	32 (23.4)	0.01 *
	Yes	3 (33.3)	105 (76.6)	

Values are presented as numbers and percentages for categorical variables, and as medians with interquartile ranges (IQR) for continuous variables. Fisher’s exact test was applied to categorical variables, and the Mann-Whitney U test was used for continuous variables. *: *p* < 0.05. FY fiscal year; IQR interquartile range; JPY Japanese yen.

**Table 2:** Factors Contributing to Unpaid Medical Expense, n = 153.

Variable		Odds Ratio (95% CI)	*p*
Hospital Length of Stay (days)		0.93 (0.70–1.25)	0.65
High-acuity unit days		1.00 (0.78–1.28)	0.98
Billed amount per ¥10,000 (JPY)		1.01 (1.00–1.01)	0.039 *
International Repatriation	No	0.87 (0.00–99.90)	0.95
Third-party financial support (e.g., travel insurance, public subsidy)	Yes	0.12 (0.02–0.915)	0.040 *
Death upon discharge	No	0.32 (0.02–5.51)	0.43
Reason for visit to Japan	Visiting Family	1.42 (0.10–19.6)	0.80
	Tourism	0.82 (0.10–6.71)	0.86
Region	Non-Asia	1.32 (0.23–7.68)	0.76

Odds ratios were calculated using multivariate logistic regression analysis. Interaction effects with hospitalization days, high-acuity unit days, billed amount, reason for visit to Japan, and region were included in the model. Full table with interaction effects available as Supplementary Material and not shown in this table. *: *p* < 0.05. JPY Japanese yen.

## Data Availability

Data for this study cannot be shared due to the confidentiality of the patients’ personal information.

## Supplementary Materials

The following supporting information can be downloaded at: https://www.mdpi.com/article/10.3390/healthcare13222893/s1, Figure S1: Directed acyclic graph (DAG) used for model construction; Table S1: Factors Associated with Unpaid Hospital Charges.

## Abbreviations

The following abbreviations are used in this manuscript:

CI	Confidence Interval
FY	Fiscal Year
IQR	Interquartile Range
JPY	Japanese Yen

## References

[1] Money J. (2021). Globalization, international mobility and the liberal international order. Int. Aff..

[2] Walt G. (2001). Globalization and health. Med. Confl. Surviv..

[3] Zuckerman J.N. (2002). Recent developments: Travel medicine. BMJ.

[4] Martens P., Akin S.M., Maud H., Mohsin R. (2010). Is globalization healthy: A statistical indicator analysis of the impacts of globalization on health. Glob. Health.

[5] Woodward D., Drager N., Beaglehole R., Lipson D. (2001). Globalization and health: A framework for analysis and action. Bull. World Health Organ..

[6] Angelo K.M., Kozarsky P.E., Ryan E.T., Chen L.H., Sotir M.J. (2017). What proportion of international travellers acquire a travel-related illness? A review of the literature. J. Travel Med..

[7] Japan National Tourism Organization Foreign Visitor Statistics. https://www.jnto.go.jp/statistics/data/visitors-statistics/.

[8] Saeki S., Kido H., Takada T., Maeda S., Anzai S. (2025). Ten-Year Agenda in International Healthcare “Overcoming the Healthcare Barriers”: Congress Report. J. Int. Health.

[9] Nakamura Y. (2020). Importance and Necessity of Medical Interpreters from the Perspective of the Current Situation in Japan. Health Care.

[10] Kido H., Saeki S. (2025). Dissemination of Information to Foreigners in Preparation for Natural Disasters. JMA J..

[11] Saeki S., Iwata M., Tomizawa R., Minamitani K. (2022). Challenges and the potential of promoting remote medical interpreting during COVID-19. Glob. Health Med..

[12] Kido H., Saeki S., Hiraiwa M., Yasunaga M., Tomizawa R., Honda C., Fukuoka T., Minamitani K. (2024). Plain language in the healthcare of Japan: A systematic review of “plain Japanese”. Glob. Health J..

[13] Saeki S., Minamitani K., Iwaoka F., Shirai K. (2022). Perspectives of Healthcare Providers towards Remote Medical Interpreting Services in Japan. Healthcare.

[14] Saeki S., Minamitani K. (2021). The true legacy of the 2020 Tokyo Olympic Games to the 2025 World Expo: A step forward to racial equity in the Japanese healthcare system. J. Int. Health.

[15] Saeki S., Kurosawa Y., Tomiyama K., Tomizawa R., Honda C., Minamitani K. (2023). Foreign Patients Visiting the Emergency Department: A Systematic Review of Studies in Japan. JMA J..

[16] Ministry of Health, Labour and Welfare Survey on the Conditions of Accepting Foreign Patients at Medical Institutions. https://www.mhlw.go.jp/stf/newpage_41976.html.

[17] Ministry of Health Labour and Welfare (2024). 202306013A A Study to Assess the Actual State of Unpaid Bills for Foreign Patients at Emergency Medical Institutions and to Examine Countermeasures.

[18] Saeki S. (2025). Universal health coverage for immigrants in Japan. Lancet Reg. Health West. Pac..

[19] Khin Y.P., Owusu F.M., Nawa N., Surkan P.J., Fujiwara T. (2025). Barriers and facilitators for healthcare access among immigrants in Japan: A mixed methods systematic review and meta-synthesis. Lancet Reg. Health West. Pac..

[20] Ikegami N., Yoo B.K., Hashimoto H., Matsumoto M., Ogata H., Babazono A., Watanabe R., Shibuya K., Yang B.M., Reich M.R. (2011). Japanese universal health coverage: Evolution, achievements, and challenges. Lancet.

[21] Inoue M., Kachi Y. (2017). Should co-payments for financially deprived patients be lowered? Primary care physicians’ perspectives using a mixed-methods approach in a survey study in Tokyo. Int. J. Equity Health.

[22] Yasukawa K., Sawada T., Hashimoto H., Jimba M. (2019). Health-care disparities for foreign residents in Japan. Lancet.

[23] Saeki S., Minamitani K., Muraki I., Shingaki T., Nagura K., Nakata K., Iso H. (2022). Defining Foreign Patients as ‘Visitors’ and ‘Residents’ in Japanese Medical Facilities: Difficulties in the Collection of Adequate Data. J. Epidemiol..

[24] Saeki S., Taniguchi H., Arahori H., Kitabatake Y., Ozono K. (2022). An International Aircraft Transport of a Neonate From Georgia to Japan. Cureus.

[25] Brockhus L., Eich A.S., Exadaktylos A., Jachmann A., Klukowska-Rotzler J. (2021). Repatriations of Ill and Injured Travelers and Emigrants to Switzerland: A Retrospective Analysis at a Tertiary Emergency Department from 2013–2018. Int. J. Environ. Res. Public Health.

[26] Akimoto M., Saeki S., Kiyomoto Y., Takeshima H., Higuchi N., Mori T., Osanai Y., Hinohara C., Inagaki T. (2025). Early discharge and international air transport of a traveler with exacerbating comorbidities and pneumomediastinum: A case report. Trop. Med. Health.

[27] Irvin C.B., Fox J.M., Pothoven K. (2003). Financial impact on emergency physicians for nonreimbursed care for the uninsured. Ann. Emerg. Med..

[28] Mylius M. (2016). Hospitalization of Migrants without Health Insurance: An Explorative Study of Hospital Healthcare in Lower Saxony, Berlin and Hamburg. Gesundheitswesen.

[29] Kishi M., Ihara I., Kusano T., Kino M., Baba T., Matsuoka T., Yukioka M. (2018). Questionnaire on the actual state of emergency medical care for foreign visitors in Osaka Prefecture. Osaka Kyukyu.

[30] Kishi M., Kusano T., Ihara I., Baba T., Matsuoka T., Kino M. (2020). Questionnaire on the actual state of emergency medical care for foreign visitors in Osaka Prefecture—An update. Osaka Kyukyu.

[31] Kainuma M., Yasunaga Y., Asano T., Mitsui H. (2019). Current Status of Foreign Patients Visiting Our Hospital for Emergency Care. Jpn. J. Hosp. Gen. Med..

[32] Shimoyama K., Azuma K., Oda J. (2020). Analysis of severe foreign patients transported to our emergency department. J. Jpn. Soc. Emerg. Med..

[33] Suzaki M., Miyauchi M., Ohara T., Wakakuri H., Kirinoki S., Onodera N., Hyodo H., Kawai M., Yokota H., Yasutake M. (2019). Current Situation and Issues of Foreign Patient Care in the ER. Jpn. J. Hosp. Gen. Med..

[34] Kokudo N., Wada K., Takei T., Matano T., Wakita T. (2025). The establishment of the Japan Institute for Health Security (JIHS): A new era in infectious disease response and research. Glob. Health Med..

[35] Hinohara C. (2024). Factors hindering hospitals from accepting foreign patients: Our issues and plans. J. Int. Health.

[36] Funato Y., Kimura A., Matsuda W., Uemura T., Kobayashi K., Sasaki R. (2024). Pain relief effect of metoclopramide vs. sumatriptan for acute migraine attack: A single-center, open-label, cluster-randomized controlled non-inferiority trial. GHM Open.

[37] Matsuda W., Kimura A., Uemura T. (2023). The reverse shock index multiplied by the Glasgow Coma Scale score can predict the need for initial resuscitation in patients suspected of sepsis. Glob. Health Med..

[38] Toyota E., Ootani N., Suzuki T., Yosikawa M., Ozawa Y., Tajima H. (1991). AN APPROACH TO TUBERCULOSIS IN NEW-COMERS FROM ASIA AND AFRICA. Kekkaku (Tuberculosis).

[39] Kanda Y. (2013). Investigation of the freely available easy-to-use software ‘EZR’ for medical statistics. Bone Marrow Transplant.

[40] R Core Team (2019). R: A Language and Environment for Statistical Computing.

[41] Ministry of Land, Infrastructure, Transport and Tourism “Survey on Medical Care for Foreign Visitors to Japan” Result. https://www.mlit.go.jp/kankocho/content/001751351.pdf.

[42] Osegawa M., Morio H., Nomoto K., Nishizawa M., Sadahiro T. (2002). Present Medical Practice and Problems in Emergency Disease in Foreign Travelers Requiring Hospital Admission. Nihon Kyukyu Igakukai Zasshi.

[43] Angell S.Y., Cetron M.S. (2005). Health disparities among travelers visiting friends and relatives abroad. Ann. Intern. Med..

[44] Ali A., Ali S., Khan S.A., Khan D.M., Abbas K., Khalil A., Manzoor S., Khalil U. (2019). Sample size issues in multilevel logistic regression models. PLoS ONE.

