# Supplementary material for: Determinants of Unpaid Hospital Charges Among Non-Resident Foreign Patients: A Retrospective Single-Center Study in Tokyo, Japan
Source: Healthcare (Basel). 2025 Nov 13;13(22):2893. doi: 10.3390/healthcare13222893 (PMC12652974; doi:10.3390/healthcare13222893)
Supplement: Supplementary file 1 [file healthcare-13-02893-s001.zip › healthcare-3911981-supplementary.pdf]

Table S1. Factors Contributing to Unpaid Medical Expense (Full).

n=153

**Variable**		**Odds Ratio (95% CI)**	**p**	
Hospital Length of Stay (days)		2.20 (0.70-6.95)	0.18	
High-acuity unit days		0.15 (0.02-1.34)	0.09	
Billed amount (JPY)		1.00 (1.00-1.00)	0.31	
International Repatriation	No	0.00 (0.00-73.80)	0.22	
Third-party financial support (e.g., travel insurance, public subsidy)	No	48.10 (1.26-1840.00)	0.037	*
Death upon discharge	No	22500.00 (1.00-502000000.00)	0.05	
Reason for visit to Japan	Overstay	— (not estimable)	1	
	Visiting Family	— (not estimable)	1	
	Residency (e.g. embassy employees)	— (not estimable)	1	
	Work/Training/Study	— (not estimable)	1	
	For treatment at another medical facility	— (not estimable)	1	
	Refugee	— (not estimable)	1	
	Tourism	— (not estimable)	1	
Region	Europe/Americas/Oceania	— (not estimable)	1	
	East Asia	— (not estimable)	1	
	Southeast Asia	— (not estimable)	1	
	Japan	— (not estimable)	1	

Odds ratios were calculated using multivariate logistic regression analysis. Interaction effects with hospitalization days, high-acuity unit days, billed amount, reason for visit to Japan, and region were included in the model. Full table with interaction effects available as supplementary material. *: $p < 0.05$.

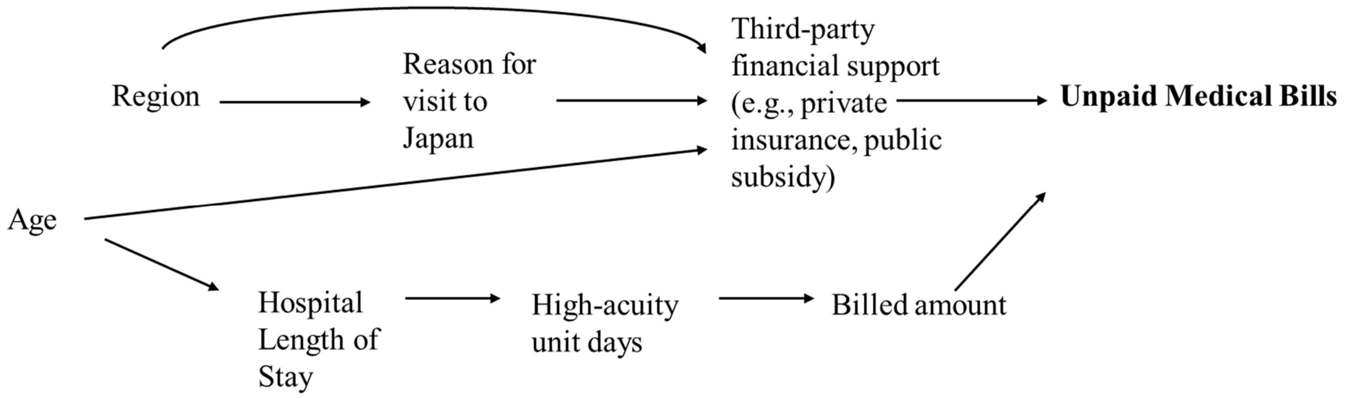

Figure S1. Directed acyclic graph (DAG) used for model construction.
